# Characterization of a microSilicon diode detector for small-field photon beam dosimetry

**DOI:** 10.1093/jrr/rraa010

**Published:** 2020-03-25

**Authors:** Yuichi Akino, Masateru Fujiwara, Keita Okamura, Hiroya Shiomi, Hirokazu Mizuno, Fumiaki Isohashi, Osamu Suzuki, Yuji Seo, Keisuke Tamari, Kazuhiko Ogawa

**Affiliations:** 1 Oncology Center, Osaka University Hospital, 2-2 (D10), Yamadaoka, Suita, Osaka 565-0871, Japan; 2 Department of Radiation Oncology, Suita Tokushukai Hospital, Suita, Osaka 565-0814, Japan; 3 Department of Radiology, Osaka University Hospital, Suita, Osaka 565-0871, Japan; 4 Department of Radiation Oncology, Osaka University Graduate School of Medicine, Suita, Osaka 565-0871, Japan; 5 Division of Health Sciences, Osaka University Graduate School of Medicine, Suita, Osaka 565-0871, Japan; 6 Department of Carbon Ion Radiotherapy, Osaka University Graduate School of Medicine, Suita, Osaka 565-0871, Japan

**Keywords:** small field dosimetry, diode detector, beam data commissioning, CyberKnife

## Abstract

This study characterized a new unshielded diode detector, the microSilicon (model 60023), for small-field photon beam dosimetry by evaluating the photon beams generated by a TrueBeam STx and a CyberKnife. Temperature dependence was evaluated by irradiating photons and increasing the water temperature from 11.5 to 31.3°C. For Diode E, microSilicon, microDiamond and EDGE detectors, dose linearity, dose rate dependence, energy dependence, percent-depth-dose (PDD), beam profiles and detector output factor (*OF*_det_) were evaluated. The *OF*_det_ of the microSilicon detector was compared to the field output factors of the other detectors. The microSilicon exhibited small temperature dependence within 0.4%, although the Diode E showed a linear variation with a ratio of 0.26%/°C. The Diode E and EDGE detectors showed positive correlations between the detector reading and dose rate, whereas the microSilicon showed a stable response within 0.11%. The Diode E and microSilicon demonstrated negative correlations with the beam energy. The *OF*_det_ of microSilicon was the smallest among all the detectors. The maximum differences between the *OF*_det_ of microSilicon and the field output factors of microDiamond were 2.3 and 1.6% for 5 × 5 mm^2^ TrueBeam and 5 mm φ CyberKnife beams, respectively. The PDD data exhibited small variations in the dose fall-off region. The microSilicon and microDiamond detectors yielded similar penumbra widths, whereas the other detectors showed steeper penumbra profiles. The microSilicon demonstrated favorable characteristics including small temperature and dose rate dependence as well as the small spatial resolution and output factors suitable for small field dosimetry.

## INTRODUCTION

Small field dosimetry has become increasingly important as advanced radiotherapy techniques including stereotactic radiotherapy (SRT), intensity-modulated radiotherapy (IMRT) and volumetric-modulated arc therapy (VMAT), are used more commonly around the world. However, accurate small field dosimetry is challenging because of various phenomena, such as lateral charged-particle disequilibrium, perturbation of the charged-particle fluence and volume averaging effects [1–4]. Several types of detectors (e.g. diodes, diamond and plastic scintillators) have been developed to minimize these effects [5–8]. P-type silicon diode detectors are often used to conduct scanning and non-scanning measurements for beam data commissioning. Compared to ionization chambers, the diode detectors have small sensitive volumes, which can minimize the volume averaging effects [9, 10]. However, diode detectors have some disadvantageous characteristics for accurate dosimetry, such as temperature, dose rate and energy dependences [11–13]. Diode detectors over-respond to low-energy photon beams because of the photoelectric effects in silicon [14, 15]. In 2008, the International Atomic Energy Agency (IAEA)/American Association of Physicists in Medicine (AAPM) proposed a formalism based on the use of correction factors [16]. The output correction factor (${k}_{Q_{\mathrm{clin}},{Q}_{\mathrm{msr}}}^{f_{\mathrm{clin}},{f}_{\mathrm{msr}}})$ corrects the detector-dependent factors for relative dosimetry of small fields. Many studies have reported that the ${k}_{Q_{\mathrm{clin}},{Q}_{\mathrm{msr}}}^{f_{\mathrm{clin}},{f}_{\mathrm{msr}}}$ factors of unshielded diode detectors are closer to unity at small field sizes than those of shielded diode detectors [1, 5, 17].

Recently, a new unshielded diode detector, the microSilicon (model 60023, PTW Freiburg, Freiburg, Germany) dosimetry diode, has been developed. This product succeeds the Diode E (model 60017, PTW) detector and is designed for electron and small-field photon beam dosimetry. In this study, we report on the characteristics of the microSilicon diode detector for small-field photon beam dosimetry.

## MATERIALS AND METHODS

### Detector characteristics

Characteristics of the detectors evaluated in this study are summarized in Supplementary Table 1, see online supplementary material. In this study, we measured small-field photon beams using the microSilicon as well as the Diode E, microDiamond (model 60019, PTW) and EDGE (model 1118, Sun Nuclear Corp., Melbourne, FL, USA) detectors for comparison. A TrueBeam STx (Varian Medical Systems, Palo Alto, CA, USA) with a HD120 multileaf collimator (MLC) was used for irradiation. For evaluating the scanning data and output factors, 6 MV photon beams of a CyberKnife G4 (Accuray, Inc., Sunnyvale, USA) collimated with fixed-type cones were also measured, and the data were compared with those measured with the Diode SRS (type 60018, PTW) acquired for commissioning. For all measurements, the Diode E, Diode SRS, microSilicon and microDiamond detector axis was parallel to the beam axis, as recommended in the IAEA Technical Reports Series no. 483 (TRS-483) [1].

### Temperature dependence

Temperature dependence was evaluated by irradiating a 6 MV photon beam with a flattening filter (WFF) by changing water temperature. The Diode E and microSilicon detectors were located at a 10 cm depth from the entrance window of the water phantom model GRI-7670A (Toyo-Medic, Tokyo, Japan), which was designed to measure lateral beams. The field size, gantry angle and source-to-surface distance (SSD) were 100 × 100 mm^2^, 270° and 90 cm, respectively. First, cold water was poured into the phantom, and measurements were repeated by replacing some of the cold water with hot water. Over the course of the experiments, the water temperature was increased from 11.5 to 31.3 °C. Because the response of the microSilicon was much higher than that of Diode E, the detector readings were normalized at 21.5 °C and the relative variations were compared.

### Dose–response linearity, and dose rate- and energy-dependences

The dose linearity, dose rate dependence and energy dependence were measured using a 1D SCANNER (Sun Nuclear) water phantom, and a RAMTEC Smart (Toyo-Medic) electrometer was used. The SSD, depth and field size were 90 cm, 10 cm, and 50 × 50 mm^2^, respectively. To evaluate the constancy of the beam output, a Farmer-type ionization chamber (model 30013, PTW) was also used for measurements. The detector readings of the Farmer-type ionization chamber were corrected by the beam quality correction factor (*k*_Q_), ion recombination correction factor (*k*_ion_), polarity correction factor (*k*_pol_) and temperature–pressure correction factor (*k*_TP_) [18]. The dose linearity was evaluated by irradiating photon beams of 3–2000 monitor units (MUs). The measurements were repeated three times for most of measurements. For <10 MU, the number of measurements ranged from 5 to 10, depending on the coefficient of variation (CV). The measurements were repeated until the CV was <1% or the number of measurements reached 10. For 3 MU, the measurements were repeated 10 times for all detectors. The correlation coefficient (*R*^2^) was calculated using a JMP Pro Software ver. 14.0 (SAS Institute Inc., Cary, NC, USA). For evaluation of dose rate dependence, the MU used for the WFF and flattening filter free (FFF) beams were 50 and 200 MU, respectively. The ranges of the dose rates were 10–250 MU/min for the 4 MV WFF, 10–600 MU/min for the 6 and 10 MV WFF, 400–1400 MU/min for the 6 MV FFF and 400–2400 MU/min for the 10 MV FFF. Because the dose rate of 400 MU/min can be used for 6 and 10 MV WFF and FFF beams, the data measured with this dose rate were used for evaluation of the energy dependence. The detector readings were divided by the dose at the detector position, calculated from the MU multiplied by the tissue-maximum-ratio (TMR) at 10 cm depth and the detector output factor (*OF*_det_), i.e. the ratio of the detector readings of the small fields to that of the machine-specific reference (msr) field size, for a 50 × 50 mm^2^ field size. The correlation between the readings/dose and TPR_20,10_ (tissue-phantom-ratio) was evaluated. Because of large variations in the response among the detectors, the readings/MU values were normalized by the value of the 6 MV WFF beam and the relative variations were evaluated.

### Scanning data and detector output factors

The percent-depth-dose (PDD), beam profiles and *OF*_det_ of the photon beams generated by the TrueBeam were measured with various detectors using a BluePhantom^2^ (IBA Dosimetry GmbH, Schwarzenbruck, Germany). The SSD was 100 cm and the depth for measurements of beam profiles and *OF*_det_ was 10 cm. The MLC field sizes for measurements of *OF*_det_ ranged from 5 × 5 mm^2^ to 220 × 220 mm^2^. For field sizes ≤ 40 × 40 mm^2^, the jaw field sizes were 2–3 mm larger than the MLC field sizes in accordance with the beam data collection protocol for the iPLAN (BrainLAB, Munich, Germany) treatment planning system. The FFF beams generated by the CyberKnife were measured using a MP3 (PTW) scanning phantom. The SSDs were 80 and 78.5 cm for collections of the scanning data and output factors, respectively, and the depths of the measurements were 10 and 1.5 cm for beam profile and output factor measurements, respectively. The beam profile data were normalized at the central axis, and the center of the profiles, calculated as the middle of the full width at half maximum (FWHM), was corrected. The lateral penumbra, the width of the 20–80% profile, was also evaluated.

### Output correction factors

For the Diode E, Diode SRS, microDiamond and EDGE detectors, the ${k}_{Q_{\mathrm{clin}},{Q}_{\mathrm{msr}}}^{f_{\mathrm{clin}},{f}_{\mathrm{msr}}}$ values provided by IAEA TRS-483 were applied to *OF*_det_ to obtain the field output factor (${\varOmega}_{Q_{\mathrm{clin}},{Q}_{\mathrm{msr}}}^{f_{\mathrm{clin}},{f}_{\mathrm{msr}}}$). For TrueBeam data, the field sizes for ${k}_{Q_{\mathrm{clin}},{Q}_{\mathrm{msr}}}^{f_{\mathrm{clin}},{f}_{\mathrm{msr}}}$ were derived from the FWHM of the crossline beam profiles measured at 10 cm depth, whereas the nominal cone diameters were used for CyberKnife data. The median FWHM of all detectors was calculated for each energy and nominal MLC field size, although WFF and FFF beams were not separately calculated because the differences of FWHM were <0.2 mm. When using detectors that are not suitable for the entire range of field sizes from machine specific reference field size (*f*_msr_) to clinical small field size (*f*_clin_), IAEA TRS-483 recommends using the intermediate field method; an ionization chamber (IC) is used for field sizes down to an intermediate field (*f*_int_), whereas the small field detectors are used only for measurements in smaller fields with re-normalization at *f*_int_. We used the *OF*_det_ data measured using a CC13 (IBA Dosimetry) ionization chamber for field sizes ≥40 × 40 mm^2^. The ${\varOmega}_{Q_{\mathrm{clin}},{Q}_{\mathrm{msr}}}^{f_{\mathrm{clin}},{f}_{\mathrm{msr}}}$ values for 5–30 mm field sizes were calculated as following:

(1) \begin{equation*} {\varOmega}_{Q_{\mathrm{clin}},{Q}_{\mathrm{msr}}}^{f_{\mathrm{clin}},{f}_{\mathrm{msr}}}={\left[\frac{M_{Q_{\mathrm{clin}}}^{f_{\mathrm{clin}}}}{M_{Q_{\mathrm{int}}}^{f_{\mathrm{int}}}}{k}_{Q_{\mathrm{clin}},{Q}_{\mathrm{int}}}^{f_{\mathrm{clin}},{f}_{\mathrm{int}}}\right]}_{det}{\left[\frac{M_{Q_{\mathrm{int}}}^{f_{\mathrm{int}}}}{M_{Q_{\mathrm{msr}}}^{f_{\mathrm{msr}}}}{k}_{Q_{\mathrm{int}},{Q}_{\mathrm{msr}}}^{f_{\mathrm{int}},{f}_{\mathrm{msr}}}\right]}_{IC} \end{equation*}

Here, ${k}_{Q_{\mathrm{int}},{Q}_{\mathrm{msr}}}^{f_{\mathrm{int}},{f}_{\mathrm{msr}}}$ of CC13 for > 40 × 40 mm^2^ field sizes are unity for both 6 and 10 MV photons [1]. The ${k}_{Q_{\mathrm{clin}},{Q}_{\mathrm{int}}}^{f_{\mathrm{clin}},{f}_{\mathrm{int}}}$ values for Diode E, microDiamond and EDGE detectors were calculated by the ${k}_{Q_{\mathrm{clin}},{Q}_{\mathrm{msr}}}^{f_{\mathrm{clin}},{f}_{\mathrm{msr}}}$ normalized at 40 × 40 mm^2^ field size (Supplementary Figure 1, see online supplementary material). Because the literature provided the ${k}_{Q_{\mathrm{clin}},{Q}_{\mathrm{msr}}}^{f_{\mathrm{clin}},{f}_{\mathrm{msr}}}$ values of the EDGE detector only for field sizes ≥8 × 8 mm^2^, extrapolated values for a 5 × 5 mm^2^ field size were used, which were similar to those reported previously [19, 20]. The ${k}_{Q_{\mathrm{clin}},{Q}_{\mathrm{int}}}^{f_{\mathrm{clin}},{f}_{\mathrm{int}}}$ of Diode E for 10 MV beams was also calculated by extrapolating the values of 6- and 8-mm square field sizes. In this study, the microDiamond detector was tentatively considered as a reference because the ${k}_{Q_{\mathrm{clin}},{Q}_{\mathrm{msr}}}^{f_{\mathrm{clin}},{f}_{\mathrm{msr}}}$ values were closest to unity among the detectors evaluated in this study.

## RESULTS

**Fig. 1.**
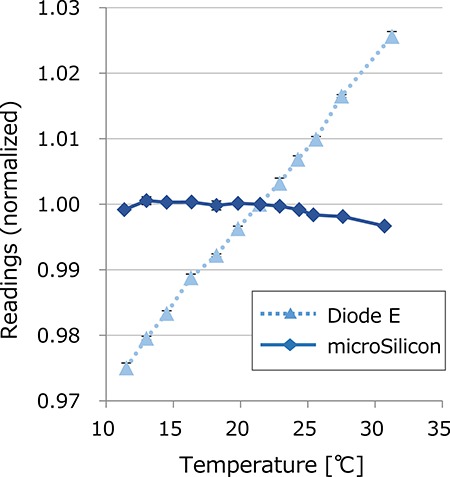
Temperature dependence of the Diode E and microSilicon detectors. The electrometer readings of the electrometer were normalized at 21.5°C.

Figure 1 illustrates the temperature dependence of the Diode E and microSilicon detectors. The Diode E exhibited a positive correlation of the response to temperature, and the difference between 11.5 and 31.3°C was 5.06%. When calculating the linear regression, the variation of the readings was 0.26%/°C. In contrast, the microSilicon diode yielded very small variations within the range from −0.33 to 0.06%.

**Fig. 2.**
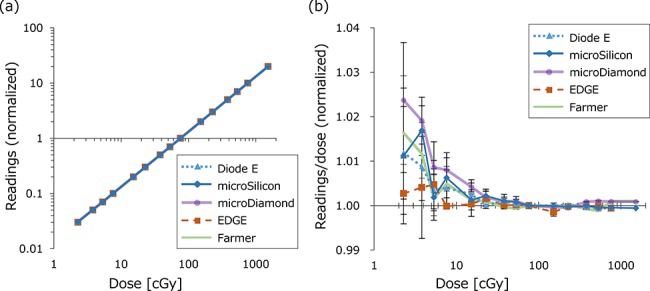
(**a**) Linearity of the detectors for 6 MV photon beams. Both horizontal and vertical axes are log scale. The values were normalized at 76.1 cGy (100 MU). (**b**) The detector readings divided by the irradiated dose, normalized at 100 MU. The points and bars represent the mean and standard deviation of measurements, respectively. Only the horizontal axis is log scale.

Figure 2a depicts the dose–response linearity for various detectors. Values were normalized at 100 MU. All detectors showed good linearity (*R*^2^ = 1.000 for all detectors). Figure 2b shows the readings of each detector divided by the irradiated dose. Most detectors, including the Farmer-type ionization chamber, showed large variations for irridation <10 cGy, likely due to the instability of the beam output. For irradiation ≥10 cGy, all detectors showed excellent linearity within 0.5%.

**Fig. 3.**
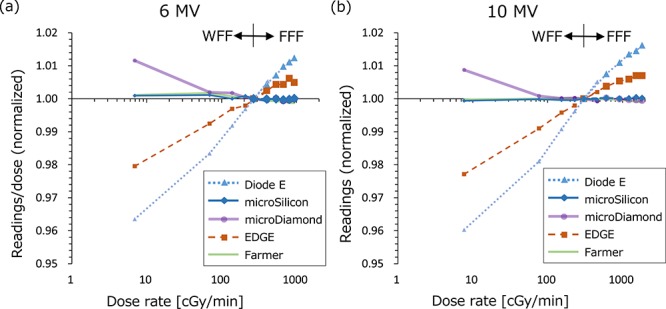
The dose rate dependence of the detectors for (**a**) 6 MV and (**b**) 10 MV with flattening filter (WFF) and flattening filter free (FFF) beams. The detector readings divided by the irradiated dose were normalized at 400 MU/min dose rate. The small and large points represent the WFF and FFF beams, respectively.

In Fig. 3, the detector readings divided by irradiated dose (nC/cGy) normalized at 400 MU/min dose rate were plotted against the dose rate. For all measurements, the CV of measurements was within 0.2%. For 6 MV and 10 MV photon beams, the values of the WFF and FFF beams were plotted in the same figure, which clearly shows the dose rate dependence of the Diode E and EDGE detectors, whereas the microSilicon showed a stable response. Because the horizontal axis is log scale, the linear plots represent the logarithmic correlation between the dose rate and the detector readings. The Farmer-type ionization chamber with corrections also yielded a stable response. The microDiamond demonstrated an increased response only at a 10-MU/min dose rate.

**Fig. 4.**
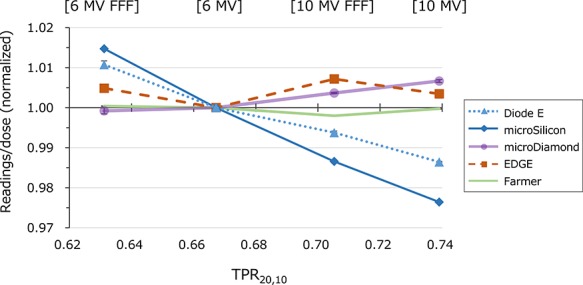
The detector readings divided by the irradiated dose normalized by the value of the 6 MV WFF beam are plotted against the TPR_20,10_ (tissue-phantom ratio) of each beam energy.

Figure 4 shows the energy dependence of each detector evaluated for a 400 MU/min dose rate. The variations of the ionization chamber, microDiamond and EDGE detectors were within 1%. On the other hand, the Diode E and microSilicon detectors exhibited negative correlations between the response and TPR_20,10_, and the microSilicon showed larger energy dependence.

**Fig. 5.**
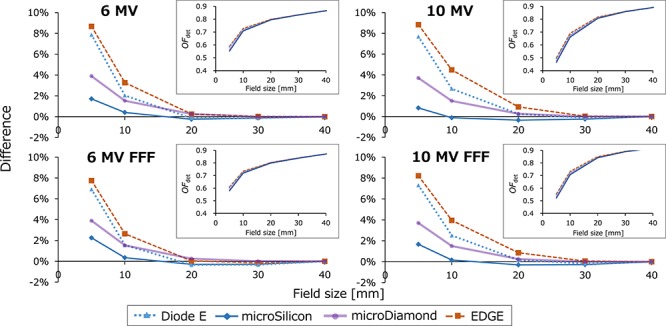
The detector output factors (*OF*_det_) of TrueBeam STx plotted against the square field sizes (insets) and the relative difference of each value from the field output factor (${\varOmega}_{Q_{\mathrm{clin}},{Q}_{\mathrm{msr}}}^{f_{\mathrm{clin}},{f}_{\mathrm{msr}}}$) of the microDiamond detector.

**Fig. 6.**
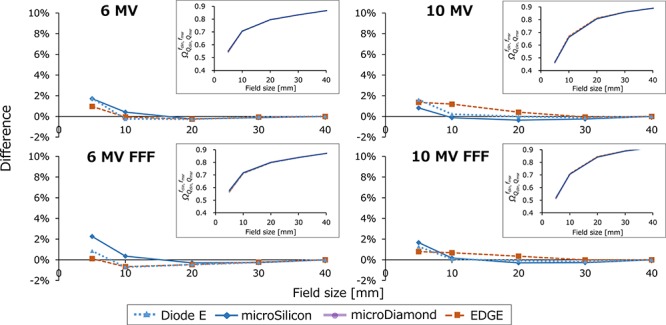
The field output factor (${\varOmega}_{Q_{\mathrm{clin}},{Q}_{\mathrm{msr}}}^{f_{\mathrm{clin}},{f}_{\mathrm{msr}}}$) of TrueBeam STx plotted against the square field sizes (insets) and the relative difference of each value from the ${\varOmega}_{Q_{\mathrm{clin}},{Q}_{\mathrm{msr}}}^{f_{\mathrm{clin}},{f}_{\mathrm{msr}}}$ of the microDiamond detector. *OF*_det_ values are plotted only for the microSilicon detector.

**Fig. 7.**
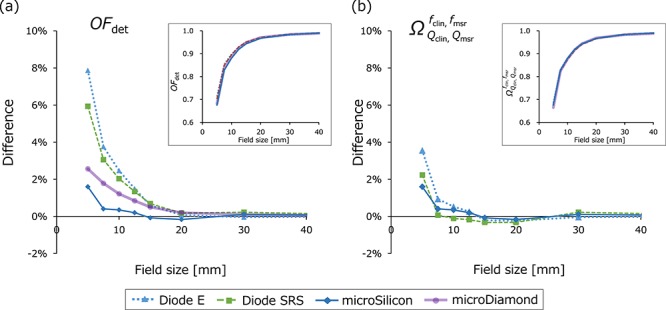
(**a**) The detector output factors (*OF*_det_) and (**b**) field output factor (${\varOmega}_{Q_{\mathrm{clin}},{Q}_{\mathrm{msr}}}^{f_{\mathrm{clin}},{f}_{\mathrm{msr}}}$) of CyberKnife plotted against the nominal cone diameter (insets) and the relative difference of each value from the ${\varOmega}_{Q_{\mathrm{clin}},{Q}_{\mathrm{msr}}}^{f_{\mathrm{clin}},{f}_{\mathrm{msr}}}$ of the microDiamond detector. *OF*_det_ values are plotted only for the microSilicon detector.

Figures 5 and 6 show the *OF*_det_ and ${\varOmega}_{Q_{\mathrm{clin}},{Q}_{\mathrm{msr}}}^{f_{\mathrm{clin}},{f}_{\mathrm{msr}}}$ values of the TrueBeam STx, respectively. In Fig. 5, the *OF*_det_ are shown in insets, and the relative differences from the ${\varOmega}_{Q_{\mathrm{clin}},{Q}_{\mathrm{msr}}}^{f_{\mathrm{clin}},{f}_{\mathrm{msr}}}$ values of the microDiamond were plotted. For all measurements the CV was within 0.1%. For ≤ 20 × 20 mm^2^ field sizes, the microSilicon demonstrated the smallest *OF*_det_ values of all detectors, whereas the EDGE detector showed the largest values. For the 5 × 5 mm^2^ field size, the mean difference of the four beam energies were 7.4, 1.6, 3.8 and 8.4% for the Diode E, microSilicon, microDiamond and EDGE detectors, respectively. Figure 6 shows the ${\varOmega}_{Q_{\mathrm{clin}},{Q}_{\mathrm{msr}}}^{f_{\mathrm{clin}},{f}_{\mathrm{msr}}}$ values of the Diode E, microDiamond and EDGE detectors, as well as the *OF*_det_ values of the microSilicon detector in insets, and the relative difference of each value from the ${\varOmega}_{Q_{\mathrm{clin}},{Q}_{\mathrm{msr}}}^{f_{\mathrm{clin}},{f}_{\mathrm{msr}}}$ of the microDiamond is also shown. For the 5 × 5 mm^2^ field size, the mean difference of the four beam energies were 1.4 and 0.8% for the Diode E and EDGE detectors, respectively. Although the values of the microSilicon detector were not corrected by the output correction factors, values were not significantly different from the ${\varOmega}_{Q_{\mathrm{clin}},{Q}_{\mathrm{msr}}}^{f_{\mathrm{clin}},{f}_{\mathrm{msr}}}$ of other detectors. The mean difference between the *OF*_det_ of the microSilicon and the ${\varOmega}_{Q_{\mathrm{clin}},{Q}_{\mathrm{msr}}}^{f_{\mathrm{clin}},{f}_{\mathrm{msr}}}$ of the microDiamond was 1.6%. Similar results were obtained from the *OF*_det_ and ${\varOmega}_{Q_{\mathrm{clin}},{Q}_{\mathrm{msr}}}^{f_{\mathrm{clin}},{f}_{\mathrm{msr}}}$ of the CyberKnife data (Figure 7). The *OF*_det_ and ${\varOmega}_{Q_{\mathrm{clin}},{Q}_{\mathrm{msr}}}^{f_{\mathrm{clin}},{f}_{\mathrm{msr}}}$ values are listed in the Supplementary Tables 2, 3 and 4, see online supplementary material. The *OF*_det_ of the Diode E and microSilicon at 100 × 100 mm^2^ field size was ~1.01, indicating the overresponse probably because of the scattered photons with low energy. If the values were normalized at 100 × 100 mm^2^ field size, the values of these detectors at small fields would be underestimated.

**Fig. 8.**
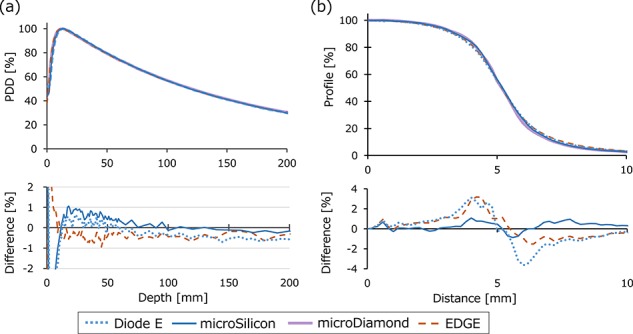
(**a**) Percent-depth-dose (PDD) and (**b**) beam profiles of the TrueBeam 6 MV photon beam with a 10 × 10 mm^2^ field size. Only the profile data in the positive axis are shown. The lower row shows the profile of the Diode E, microSilicon and EDGE detectors, with the the microDiamond detector values subtracted.

**Fig. 9.**
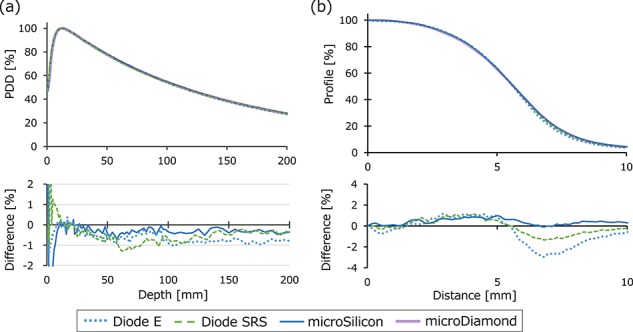
(**a**) Percent-depth-dose (PDD) and (**b**) beam profiles of the CyberKnife photon beam collimated with a 10 mm φ fixed-type cone. Only the profile data in the positive axis are shown. The lower row shows the profile of the Diode E, Diode SRS and microSilicon detectors, with the the microDiamond detector values subtracted.

Figure 8a and b depict the PDD and beam profile data of the TrueBeam 6 MV WFF beam with a field size of 10 × 10 mm^2^. The lower row presents the differences of the Diode E, microSilicon and EDGE data from the microDiamond data. The PDD demonstrate small variations within 1% in the dose fall-off region. The microSilicon detector yielded the smallest difference from the microDiamond profile data. Supplementary Table 5, see online supplementary material, shows the mean penumbra widths of both the positive and negative sides of the beam profiles. The microSilicon and microDiamond detectors showed similar penumbra widths, whereas the EDGE and Diode E detectors showed steeper penumbra profiles. Figure 9 shows the PDD and beam profiles of the CyberKnife beams. The variations of the PDD were within 1.3% in the dose fall-off region. The mean penumbra values are listed in Supplementary Table 6, see online supplementary material. The microSilicon showed similar beam profiles to those of microDiamond, whereas the Diode E and Diode SRS showed slightly steeper penumbra shape.

## DISCUSSION

In this study, we investigated the characteristics of the microSilicon diode for small-field photon beam dosimetry. Recently, Schönfeld *et al*. also reported the characteristics of the microSilicon detector [21]. In this study, we show some additional data including the temperature dependence, energy dependence and evaluation of the smaller fields of the CyberKnife collimated by cones. We demonstrated that the microSilicon detector yields stable responses in a wide range of water temperatures, whereas the Diode E detector showed a 0.26%/°C temperature-dependent response variation. For the microDiamond detector, Akino *et al*. previously reported a small variation in response, within 0.7%, in the temperature range 4–41°C [6], whereas the plastic scintillator is reported to show a temperature-dependent response [22]. Although the impact on the data measured in a water phantom may not be large, a temperature-independent detector will provide more stable data. The microSilicon also exhibited very small variation against the dose rate, although energy dependent variations of the response were observed. In contrast, the Diode E, EDGE and microDiamond demonstrated variation with dose rate. Diode E also showed energy dependence similar to that of microSilicon, whereas the EDGE and microDiamond showed small variations of <1%. The microDiamond showed a slight increase of the response at very low dose rate. Björk *et al*. previously reported similar data for electron beams [23]. Many studies have reported the the diode and diamond detectors exhibited a dose-per-pulse (DPP) dependent response [24–27]. Schönfeld *et al*. reported that the microSilicon and microDiamond detectors showed negligible DPP dependence, whereas the Diode E showed a DPP-dependent increase in response of up to 3% [21]. According to the vendor-provided information, the MU-per-pulse of the photon beams generated by the TrueBeam linear accelerator does not depend on the dose rate settings (personal communication). Therefore, the dose rate dependence observed in this study was not affected by the DPP dependence of the detectors. The energy dependence shown in Fig. 4 may be affected by DPP. With the setting of the measurements, the DPP values were 0.546, 0.214, 1.034 and 0.233 mGy/pulse for 6 MV FFF, 6 MV WFF, 10 MV FFF and 10 MV WFF beams, respectively. Although the Diode E and microSilicon detectors exhibited linear correlation between the TPR_20,10_ and the response, the DPP values were not linearly correlated with TPR_20,10_, indicating that the energy-dependent variations observed in this study were not only due to the DPP.

To evaluate the output factor measurement uncertainties with very small field size ≤10 mm, the *OF*_det_ values measured with the microSilicon were compared to the ${\varOmega}_{Q_{\mathrm{clin}},{Q}_{\mathrm{msr}}}^{f_{\mathrm{clin}},{f}_{\mathrm{msr}}}$ values measured with other detectors. Because the microSilicon detector is a brand-new product, its ${k}_{Q_{\mathrm{clin}},{Q}_{\mathrm{msr}}}^{f_{\mathrm{clin}},{f}_{\mathrm{msr}}}$ factors were not included in the IAEA TRS-483. Nevertheless, the *OF*_det_ values of the microSilicon were very close to the ${\varOmega}_{Q_{\mathrm{clin}},{Q}_{\mathrm{msr}}}^{f_{\mathrm{clin}},{f}_{\mathrm{msr}}}$ measured with other detectors. This finding is likely due to the smaller density of the entrance window and thinner water-equivalent depth of the sensitive diode disk from the detector tip, which results in less perturbation of the electron fluence. According to the vendor-provided data, the total area densities of the entrance window of the Diode E and microSilicon detectors are 140 and 92 mg/cm^2^, respectively. Schönfeld *et al*. reported the ${k}_{Q_{\mathrm{clin}},{Q}_{\mathrm{msr}}}^{f_{\mathrm{clin}},{f}_{\mathrm{msr}}}$ factors of the microSilicon determined using a W1 plastic scintillator as a reference. They reported ${k}_{Q_{\mathrm{clin}},{Q}_{\mathrm{msr}}}^{f_{\mathrm{clin}},{f}_{\mathrm{msr}}}$ values of ~0.96 for 0.55 cm effective field size [21]. Further investigations using Monte Carlo calculations will be needed to determine the optimal values. Akino *et al*. previously collected beam data for Novalis Tx (BrainLAB, Munich, Germany) from multiple institutions and reported large inter-institutional variability of the *OF*_det_ [28]. If the primary causes of the variations are not machine-specific but detector-dependent, detectors with ${k}_{Q_{\mathrm{clin}},{Q}_{\mathrm{msr}}}^{f_{\mathrm{clin}},{f}_{\mathrm{msr}}}$ values close to unity would contribute to reducing the inter-institutional variations of the dose delivered to patients.

For PDD measurements of small fields, the variation among detectors was within ±1% in the dose fall-off region. The beam profiles measured with microSilicon demonstrated a similar profile shape to that measured with the microDiamond, whereas the EDGE and Diode E showed steeper penumbrae, probably due to the smaller detector size, as shown in Supplementary Table 1. In this study, the orientation dependence of the detector was not evaluated. Because the thickness of the sensitive diode disk of the microSilicon is 18 μm, a very steep profile will be obtained if the disk is placed parallel to the beam axis. With such measurements, however, scattering and effects due to stem and cable irradiation will result in asymmetric profiles, as reported elsewhere [29]. The IAEA TRS-483 also recommended that the diode detector axis should be parallel to the beam axis [1].

Although the microSilicon detector showed characteristics suitable for small field dosimetry, the detector exhibited over-response for large field measurements (data not shown). When the *OF*_det_ of the 6 MV WFF beam was re-normalized at the 40 × 40 mm^2^ field size to the value measured with a CC13 ionization chamber, the relative differences of the *OF*_det_ of the 100 × 100 mm^2^ field size relative to the value of CC13 were 1.2, 0.9, 0.3 and −0.3% for the Diode E, microSilicon, EDGE and microDiamond, respectively. For the 220 × 220 mm^2^ field size, the relative differences were 4.6, 4.0, 2.6 and −0.2%, respectively. For middle–large field sizes, ionization chambers provide more reliable *OF*_det_ values. The microDiamond has shown excellent characteristics, such as the small spatial resolution and stable response against the beam energy, dose rate and temperature [6, 27, 30–32], although the cost is higher than for diodes.

## CONCLUSION

We investigated the microSilicon diode detector’s characteristics for small-field photon beam dosimetry. The microSilicon detector yielded smaller variations with water temperature and dose rate compared to the Diode E. The scatter factor and scanning data also indicated that the microSilicon detector provides appropriate data for small field dosimetry. With careful application, the microSilicon provides appropriate data for small field dosimetry.

## Supplementary Material

Suppl_Figure_1_rraa010

Supplementary_tables_R1_rraa010
