# Supplementary figures and images for: Characterization of a microSilicon diode detector for small-field photon beam dosimetry
Source: J Radiat Res. 2020 Mar 25;61(3):410–8. doi: 10.1093/jrr/rraa010 (PMC7299273; doi:10.1093/jrr/rraa010)

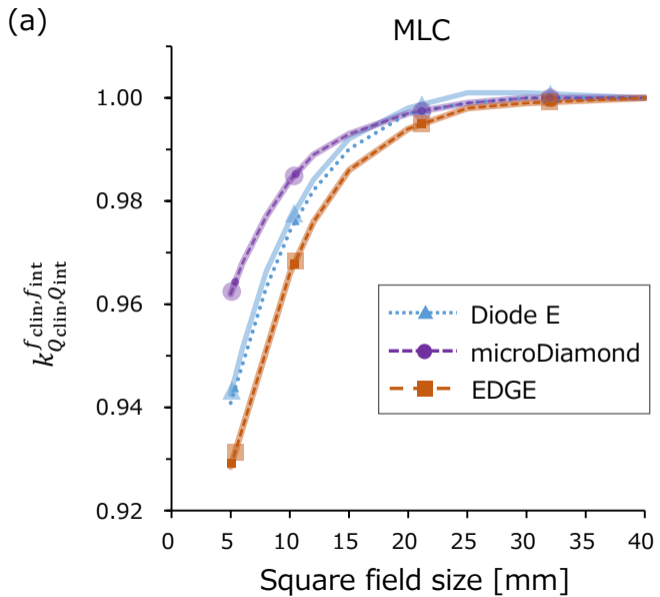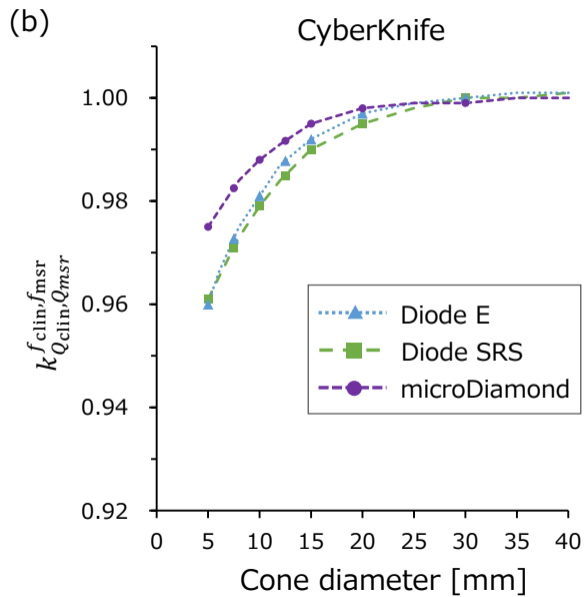

Supplement: Suppl_Figure_1_rraa010 [file suppl_figure_1_rraa010.pdf]
