# Supplementary material for: Characterization of a microSilicon diode detector for small-field photon beam dosimetry
Source: J Radiat Res. 2020 Mar 25;61(3):410–8. doi: 10.1093/jrr/rraa010 (PMC7299273; doi:10.1093/jrr/rraa010)
Supplement: Supplementary_tables_R1_rraa010 [file supplementary_tables_r1_rraa010.docx]

Supplementary table 1. Detectors’ characteristics.

| Detector | | Diode E | Diode SRS | microSilicon | microDiamond | EDGE |
| --- | --- | --- | --- | --- | --- | --- |
| Model | | 60017 | model 60018 | 60023 | 60019 | 1118 |
| Vendor | | PTW | PTW | PTW | PTW | Sun Nuclear |
| Type | | Unshielded diode | Unshielded diode | Unshielded diode | Diamond | Shielded diode |
|  |  |  |  |  |  |  |
| Sensitive volume | Area | 1.13 mm φ disk | 1.13 mm φ disk | 1.5 mm φ disk | 2.2 mm φ disk | 0.8 × 0.8 mm^2^ |
|  | Thickness | 30 μm | 250 μm | 18 μm | 1 μm | 30 μm |
|  |  |  |  |  |  |  |
| Entrance window | Density | 140 mg/cm^2^ | 140 mg/cm^2^ | 92 mg/cm^2^ | 101 mg/cm^2^ | - |
|  | Thickness ^*^ | 1.33 mm | 1.31 mm | 0.9 mm | 1.0 mm | 0.5 mm |
|  | Material | RW3 (0.3 mm),  1.045 g/cm^3^ Epoxy (0.4 mm) | RW3 (0.3 mm),  Epoxy (0.27 mm) | RW3 (0.3 mm),  Epoxy (0.48 mm),  Aluminum (0.01 mm) | RW3 (0.3 mm),  Epoxy (0.6 mm),  Aluminum (0.01 mm) | Brass (0.13 mm) |

* Water-equivalent thickness from the top of the entrance window to the reference point for photon beam dosimetry.

Supplementary table 2. Detector output factors and field output factors of TrueBeam STx 6 MV photon beams with flattening filter.

| FS [mm] | *OF*_det_ | | | |  | $\Omega_{Q_{\mathrm{clin}},Q_{\mathrm{msr}}}^{f_{\mathrm{clin}},f_{\mathrm{msr}}}$ | | |
| --- | --- | --- | --- | --- | --- | --- | --- | --- |
|  | Diode E | microSilicon | microDiamond | EDGE |  | Diode E | microDiamond | EDGE |
| 5 | 0.585 | 0.551 | 0.563 | 0.589 |  | 0.551 | 0.542 | 0.547 |
| 10 | 0.719 | 0.708 | 0.716 | 0.728 |  | 0.703 | 0.705 | 0.705 |
| 20 | 0.795 | 0.794 | 0.798 | 0.798 |  | 0.794 | 0.796 | 0.794 |
| 30 | 0.832 | 0.832 | 0.833 | 0.834 |  | 0.833 | 0.833 | 0.833 |
| 40 | 0.866 | 0.866 | 0.866 | 0.866 |  | 0.866 | 0.866 | 0.866 |
| 60 | 0.922 | 0.922 | 0.919 | 0.920 |  | 0.922 | 0.919 | 0.920 |
| 80 | 0.972 | 0.970 | 0.963 | 0.967 |  | 0.972 | 0.963 | 0.967 |
| 100 | 1.012 | 1.009 | 0.997 | 1.003 |  | 1.012 | 0.997 | 1.003 |

Abbreviations: FS, field size; *OF*_det_, field output factor; $\Omega_{Q_{\mathrm{clin}},Q_{\mathrm{msr}}}^{f_{\mathrm{clin}},f_{\mathrm{msr}}}$, field output factor.

Note: The *OF*_det_ values were normalized at 40 × 40 mm^2^ field size by the value of CC13 to evaluate the values at small fields appropriately. Field output factors were calculated only for field sizes <40 mm.

Supplementary table 3. Detector output factors and field output factors of TrueBeam STx 10 MV photon beams with flattening filter.

| FS [mm] | *OF*_det_ | | | |  | $\Omega_{Q_{\mathrm{clin}},Q_{\mathrm{msr}}}^{f_{\mathrm{clin}},f_{\mathrm{msr}}}$ | | |
| --- | --- | --- | --- | --- | --- | --- | --- | --- |
|  | Diode E | microSilicon | microDiamond | EDGE |  | Diode E | microDiamond | EDGE |
| 5 | 0.496 | 0.464 | 0.477 | 0.501 |  | 0.468 | 0.460 | 0.467 |
| 10 | 0.680 | 0.661 | 0.672 | 0.692 |  | 0.663 | 0.662 | 0.670 |
| 20 | 0.811 | 0.806 | 0.811 | 0.817 |  | 0.809 | 0.809 | 0.812 |
| 30 | 0.859 | 0.858 | 0.860 | 0.860 |  | 0.859 | 0.860 | 0.860 |
| 40 | 0.890 | 0.890 | 0.890 | 0.890 |  | 0.890 | 0.890 | 0.890 |
| 60 | 0.937 | 0.937 | 0.935 | 0.935 |  | 0.937 | 0.935 | 0.935 |
| 80 | 0.977 | 0.977 | 0.971 | 0.972 |  | 0.977 | 0.971 | 0.972 |
| 100 | 1.010 | 1.009 | 1.000 | 1.002 |  | 1.010 | 1.000 | 1.002 |

Abbreviations: FS, field size; *OF*_det_, field output factor; $\Omega_{Q_{\mathrm{clin}},Q_{\mathrm{msr}}}^{f_{\mathrm{clin}},f_{\mathrm{msr}}}$, field output factor.

Note: The *OF*_det_ values were normalized at 40 × 40 mm^2^ field size by the value of CC13 to evaluate the values at small fields appropriately. Field output factors were calculated only for field sizes <40 mm.

Supplementary table 4. Detector output factors and field output factors of CyberKnife 6 MV photon beams with flattening filter.

| FS [mm] | *OF*_det_ | | | |  | $\Omega_{Q_{\mathrm{clin}},Q_{\mathrm{msr}}}^{f_{\mathrm{clin}},f_{\mathrm{msr}}}$ | | |
| --- | --- | --- | --- | --- | --- | --- | --- | --- |
|  | Diode E | microSilicon | microDiamond | EDGE |  | Diode E | microDiamond | EDGE |
| 5 | 0.717 | 0.704 | 0.682 | 0.675 |  | 0.688 | 0.677 | 0.665 |
| 10 | 0.856 | 0.850 | 0.839 | 0.828 |  | 0.832 | 0.825 | 0.825 |
| 20 | 0.899 | 0.895 | 0.888 | 0.880 |  | 0.882 | 0.876 | 0.877 |
| 30 | 0.931 | 0.930 | 0.925 | 0.919 |  | 0.920 | 0.916 | 0.917 |
| 40 | 0.949 | 0.950 | 0.949 | 0.943 |  | 0.942 | 0.941 | 0.944 |
| 60 | 0.969 | 0.970 | 0.971 | 0.967 |  | 0.966 | 0.966 | 0.969 |
| 80 | 0.983 | 0.985 | 0.984 | 0.984 |  | 0.983 | 0.985 | 0.983 |
| 100 | 1.000 | 1.000 | 1.000 | 1.000 |  | 1.000 | 1.000 | 1.000 |

Abbreviations: FS, field size; *OF*_det_, field output factor; $\Omega_{Q_{\mathrm{clin}},Q_{\mathrm{msr}}}^{f_{\mathrm{clin}},f_{\mathrm{msr}}}$, field output factor.

Supplementary table 5. The penumbra widths of the TrueBeam STx 6 MV photon beams (mm).

| FS | Energy | Detectors | | | |
| --- | --- | --- | --- | --- | --- |
|  |  | Diode E | microSilicon | microDiamond | EDGE |
| 10 × 10 mm^2^ | 6 MV | 2.11 | 2.37 | 2.40 | 2.21 |
|  | 6 MV FFF | 1.99 | 2.22 | 2.30 | 2.13 |
|  | 10 MV | 2.82 | 3.04 | 3.10 | 2.93 |
|  | 10 MV FFF | 2.60 | 2.81 | 2.84 | 2.73 |
|  |  |  |  |  |  |
| 20 × 20 mm^2^ | 6 MV | 2.40 | 2.72 | 2.69 | 2.51 |
|  | 6 MV FFF | 2.25 | 2.52 | 2.55 | 2.37 |
|  | 10 MV | 3.38 | 3.75 | 3.74 | 3.56 |
|  | 10 MV FFF | 3.10 | 3.43 | 3.44 | 3.27 |

Abbreviations: FS, field size; FFF, flattening-filter-free

Note: Mean values of right and left penumbrae are listed

Supplementary table 6. The penumbra widths of the CyberKnife 6 MV photon beams (mm).

| FS | Detectors | | | |
| --- | --- | --- | --- | --- |
|  | Diode E | Diode SRS | microDiamond | microSilicon |
| 5 mm φ | 2.33 | 2.41 | 2.54 | 2.56 |
| 7.5 mm φ | 2.65 | 2.72 | 2.93 | 2.86 |
| 10 mm φ | 3.02 | 3.06 | 3.26 | 3.21 |
| 30 mm φ | 3.53 | 3.67 | 3.84 | 3.90 |
| 60 mm φ | 6.27 | 6.61 | 6.87 | 6.82 |

Abbreviations: FS, field size; FFF, flattening-filter-free

Note: Mean values of right and left penumbrae are listed
